# Pain in School: Patterns of Pain-Related School Impairment among Adolescents with Primary Pain Conditions, Juvenile Idiopathic Arthritis Pain, and Pain-Free Peers

**DOI:** 10.3390/children3040039

**Published:** 2016-11-30

**Authors:** Anna Monica Agoston, Laura S. Gray, Deirdre E. Logan

**Affiliations:** 1 Division of Perioperative and Pain Medicine, Department of Anesthesia, Boston Children’s Hospital, Pain Treatment Service, 333 Longwood Ave., Boston, MA 02115, USA; monicaagost@gmail.com; 2 Department of Psychiatry, Harvard Medical School, Boston, MA 02115, USA; 3 Division of Anesthesiology, Sedation and Perioperative Medicine, Children’s National Health System, Washington, DC 20010, USA; deirdre.logan@childrens.harvard.edu; 4 The George Washington University School of Medicine, Washington, DC 20037, USA

**Keywords:** chronic pain, child and adolescent, school functioning

## Abstract

Children with chronic pain frequently experience impairment in the school setting, but we do not yet understand how unique these struggles are to children with primary pain conditions compared to peers with disease-related pain or those without chronic pain symptoms. The objective of this study is to examine school functioning, defined as school attendance rates, overall quality of life in the school setting, and school nurse visits among adolescents with primary pain conditions, those with juvenile idiopathic arthritis (JIA)-related pain, and healthy peers. Two hundred and sixty adolescents participated in the study, including 129 with primary pain conditions, 61 with JIA, and 70 healthy comparison adolescents. They completed self- and parent-reported measures of school function. Findings show that as a group, youth with primary pain conditions reported more school absences, lower quality of life in the school setting, and more frequent school nurse visits compared to both adolescents with JIA-related pain and healthy peers. We conclude that compared to those who experience pain specific to a disease process, adolescents with primary pain conditions may face unique challenges in the school setting and may require more support to help them succeed in school in spite of pain.

## 1. Introduction

School functioning is known to be impaired in adolescents with chronic pain [1,2,3,4,5,6,7]. A 2008 study by Logan and colleagues [4] demonstrated that adolescents with chronic pain without clear etiology, or “primary pain conditions” [8], missed significant amounts of school, with close to half of those sampled missing at least a quarter of school days. Other areas of school function including academic performance, engagement, attention to classwork, and keeping up with activities may be affected as well. Few studies have examined utilization of medical resources at school such as frequency of nurse visits; this is also important to understand, as it is both an indicator of health service use and of time spent disengaged from the classroom. 

Although rates of school absences in pediatric primary pain conditions have been shown to be higher than rates for most other chronic illnesses [2,9], few direct comparisons have been made with chronic diseases that include a significant pain component. Previous studies suggest that teachers and school administrators lack an understanding of primary pain conditions and of the biopsychosocial framework that explains such pain experiences [10,11]. In vignette-based studies, the absence of a clear, organic explanation for pain symptoms has been linked to more negative responses to pain complaints and less willingness to provide classroom accommodations [12]. Such attributions may be a perpetuating factor in the pain-related school impairment of youth with primary conditions. Peer reactions may also differ to these varied types of pain experience, which could further affect the child’s ability to cope with pain in school. In addition, children’s own self-perceptions around their pain and their attributions of its causes may also differ by the type of pain experience and may in turn affect their school functioning. However, the current literature does not adequately explore differences in youth with primary pain compared to those with disease-related pain symptoms, whose pain may be more easily understood by school personnel due to its more straightforward biomedical nature.

To date, the existing literature is contradictory. One study comparing youth with headache to those with disease-related pain (i.e., juvenile idiopathic arthritis (JIA), sickle cell disease) found no differences in rates of school attendance [13]. Another study comparing youth with JIA to youth with primary musculoskeletal pain found that patients with non-disease-related pain reported more school impairment as indicated by more worries about school and the presence of learning disabilities [14]. Additional studies are needed to clarify the relationship between school functioning and chronic pain in children with primary pain vs. pain due to a specific etiology and to expand the definition of school functioning beyond simply school attendance rates. There is also a dearth of research directly comparing school functioning in children with chronic pain with their healthy peers. The lack of available normative rates of school absence, nurse visits, and other indicators of school functioning makes it difficult to interpret reports of these issues in youth with chronic pain. Comparing these youth to healthy peers of a similar age and sex is critical for understanding the extent and implications of impaired school functioning in children with primary pain conditions. 

Our study investigated whether children with chronic pain of an unknown origin differed in reports of school functioning compared to children with pain due to a specific disease process, in this case JIA, and to pain-free peers. Clarifying the picture of school functioning with regard to absences, quality of life in the school setting, and utilization of health care services (e.g., number of nurse visits in children with primary pain conditions) compared to other populations will improve our understanding of risk and protective factors for school outcomes in children with primary chronic pain.

## 2. Material and Methods

### 2.1. Participants

Three groups of adolescents aged 12–19 years were recruited, a primary chronic pain (“PCP”) group, a group diagnosed with juvenile idiopathic arthritis (“JIA”), and a comparison group of healthy adolescents (“Healthy”).

The PCP group was made up of adolescent patients who underwent a multidisciplinary pain evaluation at tertiary pain clinic in a large, urban northeast pediatric hospital (Boston Children’s Hospital, Boston, MA, US). These patients included adolescents with physician-assigned diagnosis of functional gastrointestinal disorders (FGID), headache, or diffuse/localized musculoskeletal/neuropathic pain syndrome without clear disease etiology. Patients for the JIA group were recruited from pediatric rheumatology outpatient clinics at Boston Children’s Hospital Boston, MA, US. JIA was selected as the disease-based pain comparison group because it has been included in numerous other studies of pain-related functioning, including a few studies specifically examining school impairment and contrasting this condition to primary pain conditions [13,14,15]. Additionally, JIA is more prevalent in girls, which is a similar profile to most types of primary pain conditions. Girls were over-sampled for the Healthy group to align with the characteristics of the PCP and JIA groups.

Eligibility criteria for participants in the PCP and JIA groups included a current pain frequency of at least once a week or five days per month. JIA patients were excluded if they had any medical condition or separate chronic pain syndrome (e.g., migraine headache, functional abdominal pain disorder). Healthy participants were excluded if they had any current chronic pain problem that occurred at least once a week or five days per month, or any current chronic medical condition. Across all groups, participants were excluded if they were not in the 12–19 year age range, were unable to speak sufficient English, had any cognitive impairment or severe psychiatric disorder, or were not enrolled in a structured school setting. Mental health conditions such as anxiety or depression were not exclusionary. Youth receiving temporary homebound tutoring because of pain problems were included. 

### 2.2. Procedures

Data presented herein are drawn from a larger longitudinal study on school functioning and self-image in adolescents with pain. Institutional Review Board approval (IRB-P00000729) was obtained prior to the start of the study. All data collection occurred during the school months so that current school-year data could be collected for all participants. Adolescents in the PCP and JIA groups were identified in advance of scheduled clinic appointments and sent information about the study by mail. Participants who did not wish to be approached about the study during their clinic visit were asked to send back a stamped postcard indicating this. Those who did not opt out were approached when arriving for their clinic visit and the study was explained to them in detail. Adolescents recruited during hospital clinic visits were given the option of completing measures on paper immediately or receiving a link to complete them electronically. Healthy adolescent participants contacted the study team through information provided in advertisements for a study about health and school functioning. These advertisements were posted on the hospital website (accessed by staff and families) and on notices throughout the community. Girls were over-sampled by posting advertisements recruiting only for girls once sufficient numbers of boys were enrolled. Healthy participants completed all measures electronically. Assent was obtained from all participants under 18 in the PCP and JIA groups. Informed written consent was obtained from the participant’s parent and directly from the participant if over 18. Permission was received by the Institutional Review Board to provide healthy participants with an information form in lieu of formal written consent, since these participants were not recruited in person. All available data were included for analyses, thus some analyses differed in sample size due to missing data. 

### 2.3. Measures 

#### 2.3.1. Demographic and Medical Information

Age, sex, grade, and ethnicity were provided by parents of all participants. Diagnoses were obtained from medical records based on the evaluations at the clinical visit concurrent with study enrollment. Pain characteristics included “typical” pain intensity, assessed with a numeric rating scale, frequency of pain (measured on an eight-point scale from “Never” to “Daily or Almost Daily”) and time since pain onset.

#### 2.3.2. Self-Report of School Functioning

The Pediatric Quality of Life inventory (Peds-QL) [16] school functioning subscale is a five-item child/adolescent rating scale assessing subjective impressions of the extent to which pain interferes with school attendance and performance (e.g., “In the past 3 months, how much of a problem have you had with keeping up with school activities?”). The Peds-QL is a well-validated instrument for use with children with a variety of chronic medical conditions and demonstrates high reliability across items (α = 0.80).

#### 2.3.3. School Attendance

Parents reported the number of days that their adolescent missed school in the previous three months, a time frame that has been used in our previous studies of school function, showing good reliability. If participants arrived at school late or left early on a given day due to pain, these days were counted as 0.5 of a missed day, consistent with previous research [4,17]. Past studies have demonstrated close correlations between official school absence records and parent reports of school attendance, which tends to be more accessible information in the context of a research study [4].

#### 2.3.4. Frequency of Nurse’s Visits

Adolescents estimated the frequency of use of school medical services (e.g., visits to the nurse’s office) in the previous month. The time frame for reporting nurse visits was limited to one month in hopes of maintaining a reliable time frame for retrospective recall. 

### 2.4. Data Analytic Plan

The statistical analysis program SPSS version 23 (IBM Corp., Armonk, NY, USA) was used for all data analyses. Group differences in school functioning across diagnoses were examined using one-way analysis of variance (ANOVA), analysis of covariance (ANCOVA), and Student’s *t*-tests.

## 3. Results

### 3.1. Descriptive Findings 

One hundred and twenty-nine patients with primary pain, 61 with JIA, and 70 healthy comparison adolescents were recruited for a total of 260 participants. Participant ages ranged from 12 to 19 years (Mean (M) age = 15.25, standard deviation (SD) = 1.67), with no significant differences between groups in age (*F*(2,252) = 0.91, *p* = 0.41). The grade in school ranged from 6th to 12th grade. Further, 85.8% of respondents were Caucasian. In all three groups the majority of adolescent participants were female (70.5%), but a higher proportion of the PCP group were female (81.4%) compared to the JIA (62.3%) and Healthy (65.7%) comparison groups despite efforts to recruit comparable samples. Mothers were the parent responders for 86% of the sample. Families tended to report relatively high socio-economic status, with a mean Hollingshead score of 5.2 (5 = Clerical and Sales Worker) for mothers and 6.2 (6 = Technicians, Semiprofessionals) for fathers. Mothers had a mean of 5.9 education (6 = Standard College) and fathers had a mean of 5.6 education (5 = Partial College). Across the sample, 76.7% of parents were married, but this differed across groups (72% for the PCP group, 85.5% for the JIA group, and 78.4% for the Healthy group). 

Table 1 displays the frequency of specific primary diagnoses within each of the two pain groups. Compared to the JIA group, the primary pain group reported higher typical pain intensity ratings (PCP group typical pain M = 5.73, SD = 2.10; JIA group typical pain M = 2.47, SD = 2.16; *t* = 9.45, *p* < 0.001) and greater pain frequency (PCP group M = 5.88 where 6 = several times per week, SD = 2.00; JIA group M = 4.28 where 4 = several times per month, SD = 2.43; *t* = 8.74, *p* < 0.001). On the contrary, participants with JIA report a significantly longer time since pain onset compared to the primary pain group (mean time since pain onset in JIA group = 77.0 months, SD = 50.9 months; *t* = 6.4, *p* < 0.001). Given the significant between-group differences in pain characteristics, these variables were incorporated into subsequent group comparisons. 

### 3.2. Quality of Life in the School Setting.

**Figure 1:**
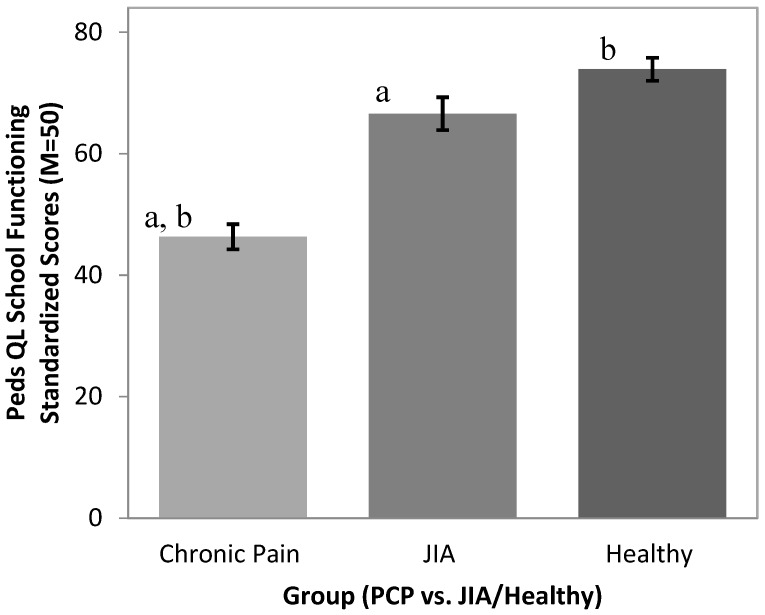
Pediatric Quality of Life inventory (Peds-QL) school function scores in Chronic Pain (PCP), juvenile idiopathic arthritis (JIA) and Healthy groups. Groups marked “a” differ from one another at *p* < 0.001; Groups marked “b” differ from one another at *p* < 0.001.

The PCP group (M = 46.32, SD = 23.42) had lower school functioning scores on the Peds-QL compared to both the JIA group (M = 66.59, SD = 20.99; *p* < 0.001) and the Healthy group (M = 73.91, SD = 16.32; *p* < 0.001) (Figure 1). Significant differences were found between groups for Peds-QL school functioning (*F*(2, 241) = 41.21, *p* < 0.001), with Tukey’s post hoc tests revealing significantly lower scores in the PCP group compared to the JIA and Healthy groups. The JIA and Healthy group means did not differ statistically. ANCOVA analyses incorporating pain characteristics revealed that group differences in Peds-QL school function scores were not accounted for by pain intensity, pain frequency, or time since pain onset. However, pain intensity did emerge as a predictor of school-related quality of life in ANCOVAs focused on the two pain groups (*F*(1,157) = 13.17, *p* < 0.001). Neither pain frequency nor time since pain onset were significant predictors of Peds-QL scores. 

### 3.3. Attendance

**Figure 2:**
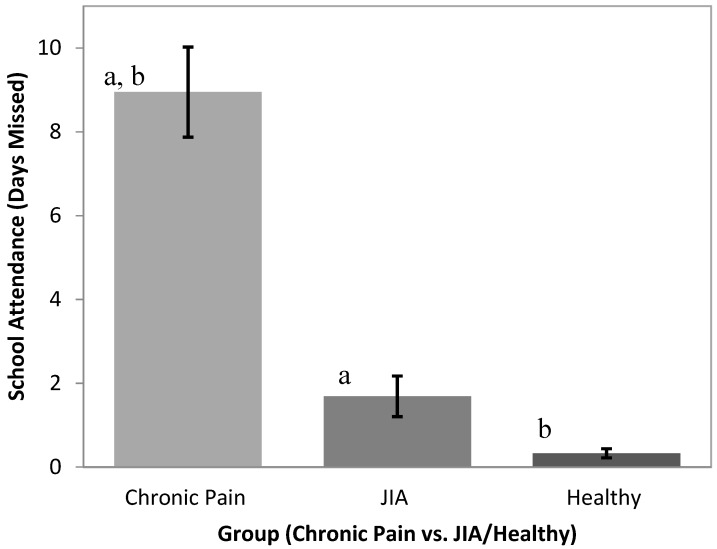
School absences in previous three months in youth in PCP, JIA, and Healthy groups. Groups marked “a” differ from one another at *p* < 0.001; Groups marked “b” differ from one another at *p* < 0.001.

The PCP group (M = 8.95, SD = 12.22) had significantly higher numbers of school days missed in the past three months compared to both the JIA group (M = 1.69, SD = 3.76; *p* < 0.001) and the Healthy group (M = 0.33, SD = 0.93; *p* < 0.001) (Figure 2). There were significant between-group differences between the number of school days missed (*F*(2,245) = 26.21, *p* < 0.001). Tukey’s post hoc tests revealed a significantly higher number of days missed in the PCP group compared to the JIA and Healthy groups. The JIA and Healthy group means did not differ statistically. ANCOVA analyses incorporating pain characteristics revealed that group differences in school attendance rates were not accounted for by pain intensity, pain frequency, or time since pain onset. No pain characteristics emerged as significant predictors of school attendance rates in these analyses. 

### 3.4. Nurse’s Office Visits

**Figure 3:**
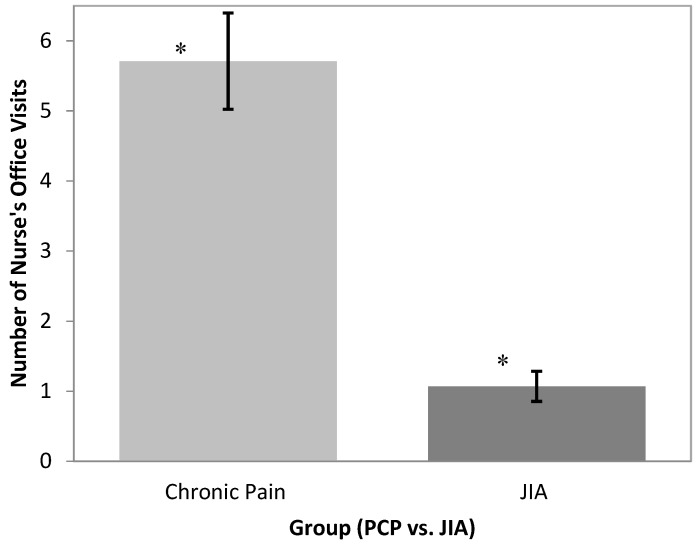
Frequency of school nurse visits in past month in PCP and JIA groups. * Groups differ significantly at *p* < 0.001.

The PCP group was found to utilize school nurse visits more often (M = 5.71, SD = 7.81) than the JIA group (M = 1.07, SD = 1.67) (see Figure 3). A Student’s *t*-test revealed a significant between-group difference between school nurse visits for the PCP vs. JIA groups (this variable was not collected for the Healthy group): *t*(170) = 4.43, *p* < 0.001. ANCOVA analyses incorporating pain characteristics revealed that group differences in the frequency of school nurse visits could be explained by differences in pain intensity between groups (*F*(1,154) = 1.65, *p* = 0.201). Pain intensity was a significant independent predictor of the frequency of nurse office visits (*F*(1,154) = 6.57, *p* < 0.05). Neither pain frequency nor time since pain onset accounted for group variation in nurse office visits. 

## 4. Discussion

Our study suggests that school functioning among youth with primary pain conditions differed significantly from that of youth with chronic pain related to JIA. Specifically, adolescents with primary pain conditions had poorer school functioning/quality of life, missed an average of nine days of school in three months compared to 1.7 days missed on average in the JIA group, and reported more nurse visits than adolescents with chronic pain due to JIA. Our results also demonstrated that youth with primary pain conditions had a lower school quality of life and missed more school than participants in a comparison group of peers without chronic pain. 

To date, studies reporting school-related outcomes in adolescents with chronic pain have suffered from a lack of context to understand the extent of school impairment among this group. 

The findings of the current study add to our knowledge regarding school functioning in adolescents with primary pain conditions by highlighting ways in which this group may experience greater levels of impairment compared to adolescents with pain due to a specific disease process and to healthy adolescents. Importantly, although pain characteristics were dissimilar across the groups in several ways, the results show that factors such as pain intensity or frequency did not fully explain group differences in school functioning, suggesting a more qualitative difference in the school experiences of youth with different types of pain conditions. 

Integrating the current findings with previous research allows us to speculate on some possible explanations for these differences. Prior studies have demonstrated that school personnel express a limited understanding of primary pain disorders and more difficulty responding in a supportive manner to pain when symptoms are not tied to a specific disease etiology [10,11]. Adolescents with primary pain conditions may consequently feel less accepted and supported at school by both peers and school staff. Other studies have indicated that these adolescents report more difficulty with peer relationships than healthy peers and are more isolated and withdrawn, less well liked, and have fewer reciprocal friendships [18]. It is possible that peers and school staff alike have difficulty understanding and acknowledging pain that does not have a straightforward familiar cause. Youth with primary pain conditions may feel invalidated in their interactions with peers and school staff and may avoid these situations, resulting in greater school absences.

In addition to differences in school attendance and overall school functioning, youth with primary pain conditions were found to utilize school health resources more often when at school compared to youth with JIA-related pain. On average, the PCP group reported visiting the nurse close to six times in the past month, compared to the one visit average reported in the JIA group. More research is needed to understand how youth are utilizing these resources and whether these resources are supporting their school functioning. For example, youth with primary chronic pain may be visiting the school nurse for an opportunity to practice active coping strategies for pain management, to obtain medication, or to avoid class time due to feeling unprepared during class or unsupported by teachers. Importantly, one must be present at school to visit the school nurse, so in some ways this behavior represents less impaired function compared to complete school absence. Future studies may explore the clinical implications of nurse visits and whether these visits are detrimental or supportive toward improving school functioning. Finally, considering our results are mixed with regard to consistency with prior studies [14,15], additional studies are needed to replicate our findings.

A major challenge to this line of research is the significant role of individual differences in efforts to foster school success among youth with chronic pain. The unique strengths and needs of each child, coupled with vast differences across school environments, require tailored school planning and make it difficult to establish uniform “best practices” regarding school accommodations for youth with primary pain conditions [19]. Nonetheless, continued research in this area plays an important role in establishing recommended approaches to addressing the needs of adolescents whose pain has led to school impairment. 

Several limitations to the study bear noting. Recruitment approaches and referral patterns in the clinic settings where the study took place may have resulted in some selection bias to the sample, limiting the comparability of the groups. Firstly, our JIA sample was recruited from rheumatology clinics where such patients are typically seen on a regular basis for ongoing monitoring, whereas the primary chronic pain patients recruited from a tertiary chronic pain clinic may have been more likely to schedule visits due to current levels of symptom distress and/or disability. Thus, our clinically-referred primary chronic pain group may represent a more severely symptomatic and/or disabled subgroup than our clinically-referred JIA group. Secondly, the Healthy group was recruited through advertisements for a study on “health and school functioning”, which may have yielded a response bias toward individuals with an interest in reporting on their school functioning, possibly due to more positive self-perception in this realm. Furthermore, the tracking of study refusals or consent without completed surveys was inconsistent across groups (i.e., better tracking occurred for the clinical samples than the healthy sample) and thus cannot be reliably reported. Another study limitation is the failure to collect data on nurse visits from the Healthy group. This was a flaw in the survey design, as such data would have informed our understanding of normal rates of this behavior. 

This study helps elucidate the relationship between school functioning and chronic pain in children with primary pain conditions compared to JIA-related pain, with possible implications for disease-related pain more broadly. By directly comparing school functioning in children with chronic pain with their healthy peers, the study also fosters increased understanding of the extent and implications of impaired school functioning in children with primary chronic pain conditions. Future studies may expand from this set of findings to advance our knowledge of risk and protective factors for school outcomes in children with chronic pain.

## Figures and Tables

**Table 1:** Frequency of primary diagnoses in primary pain (PCP) and juvenile idiopathic arthritis (JIA) groups.

PCP Group (*n* = 129)	*n* (%)
Neuropathic pain	31 (24%)
Back or neck pain	20 (15.5%)
Other myofascial/musculoskeletal pain	15 (11.6%)
Joint pain ^1^	15 (11.6%)
Functional abdominal pain	12 (9.3%)
Pelvic pain/Endometriosis ^2^	7 (5.4%)
Headache (migraine and/or tension)	5 (3.8%)
Chest pain	4 (3.1%)
Other (e.g., TMJ, coccydynia)	20 (15.5%)
**JIA Group (*n* = 61)**	***n* (%)**
Polyarticular arthritis	18 (29.5%)
Pauciarticular arthritis	9 (14.8%)
Spondyloarthropathy	16 (26.2%)
Psoriatic arthritis	12 (19.7%)
Systemic arthritis	2 (3.3%)
Enthesitis-related	2 (3.3%)
Osteomyelitis	2 (3.3%)

^1^ Of participants with joint pain as a major complaint, five had a diagnosis (current or historical) of Ehlers–Danlos syndrome but were found to have pain that was not accounted for by this diagnosis, and therefore were viewed as having a primary pain condition; ^2^ Of participants with pelvic pain as a major complaint, six had a diagnosis (current or historical) of endometriosis but were found to have pain that was not accounted for by this diagnosis, and therefore were viewed as having a primary pain condition; TMJ: temporomandibular joint disorders.
